# Supplementary material for: Molecular Shape-Preserving Au Electrode for Progesterone Detection
Source: Sensors (Basel). 2025 Mar 6;25(5):1620. doi: 10.3390/s25051620 (PMC11902543; doi:10.3390/s25051620)
Supplement: Supplementary file 1 [file sensors-25-01620-s001.zip › sensors-3475847-supplementary.pdf]

Supporting information

Utilizing Molecular Shape-Preserving Gold Electrode for Progesterone Detection

Fukuto Soyama *, Kenshin Takemura, Taisei Motomura, Wataru Iwasaki and Nobutomo Morita

Sensing System Research Center, National Institute of Advanced Industrial Science and Technology (AIST), 807-1 Shuku-Machi, Tosu, Saga 841-0052, Japan

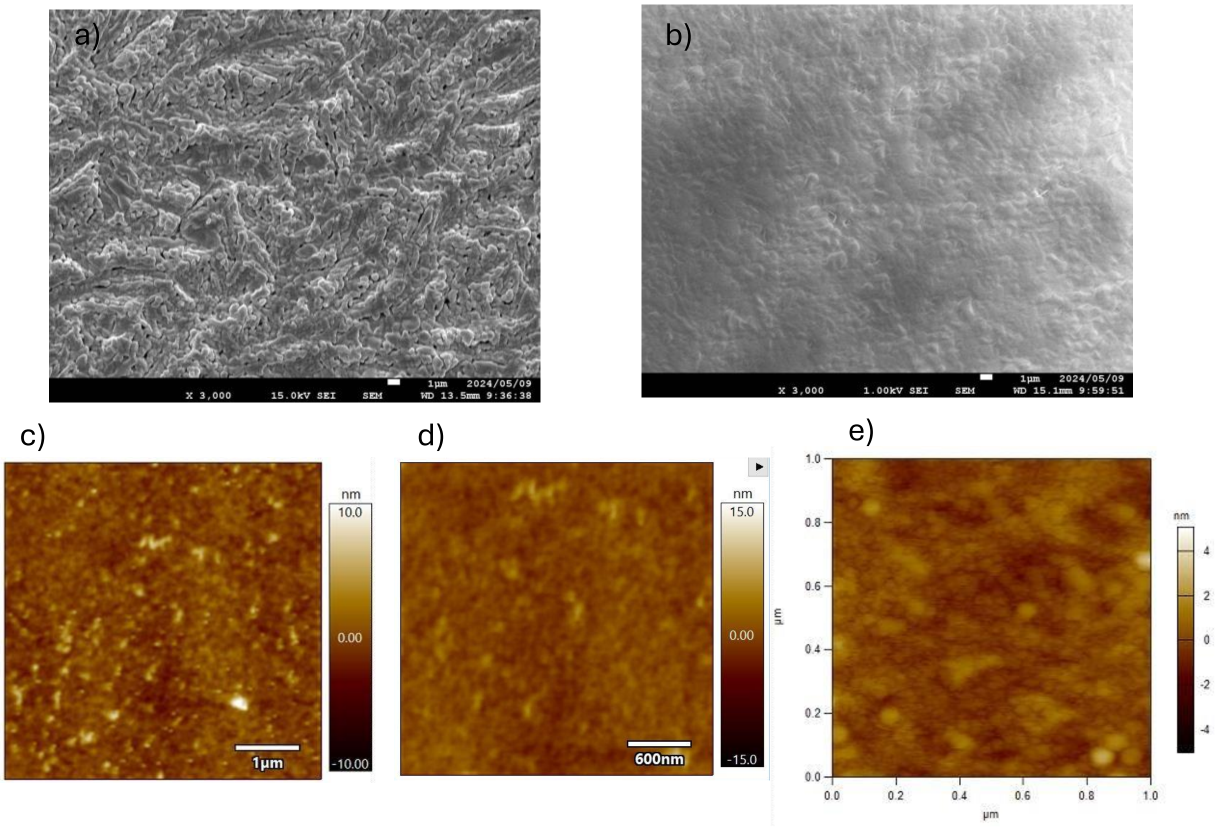

Figure S1. Analysis of the electrode surface. (a) SEM image before electrodeposition with nickel. (b) SEM image after electrodeposition with nickel. (c) AFM results at 5 µm square after electrodeposition with nickel. d) AFM results at 3 µm square after electrodeposition with nickel. (e) AFM results at 1 µm square after electrodeposition with nickel.

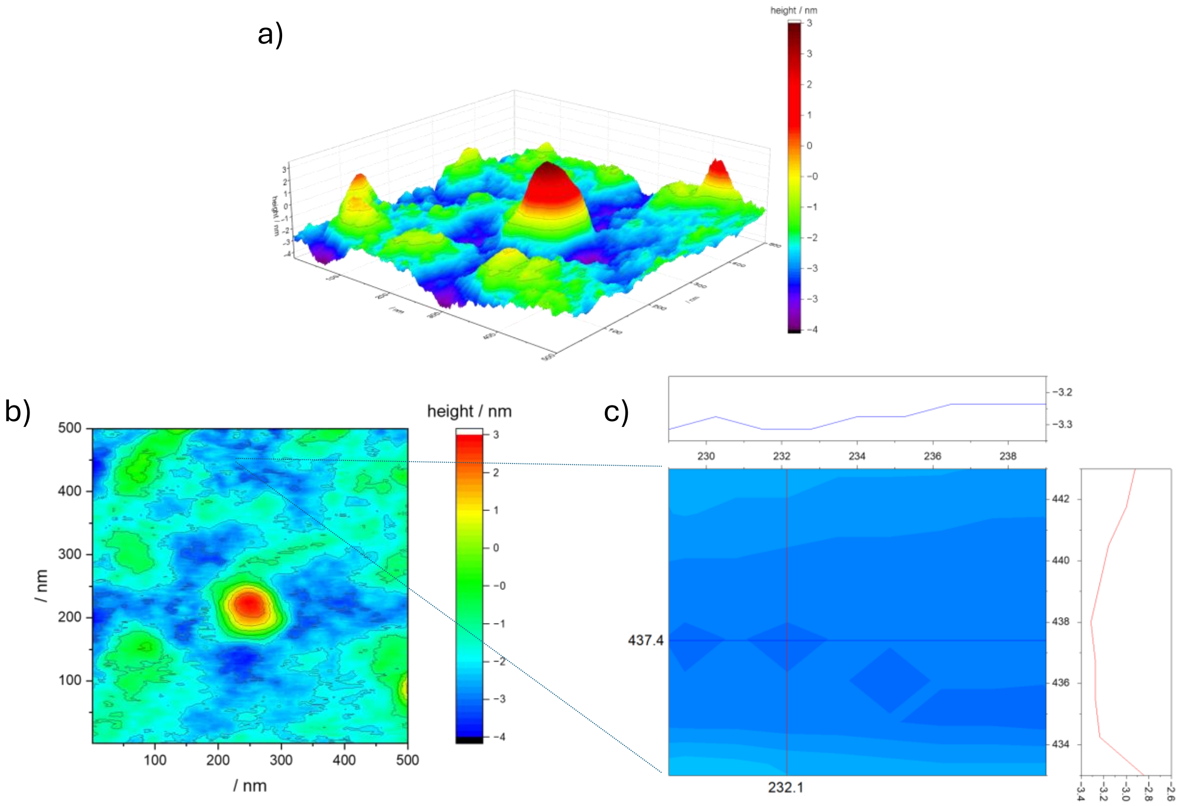

Figure S2. Analysis of the electrode surface. (a) 3D view of image analysis results based on AFM 2D data. (b) 2D contour map based on a 3D view of (a). (c) Profile of the dented area of the contour map.

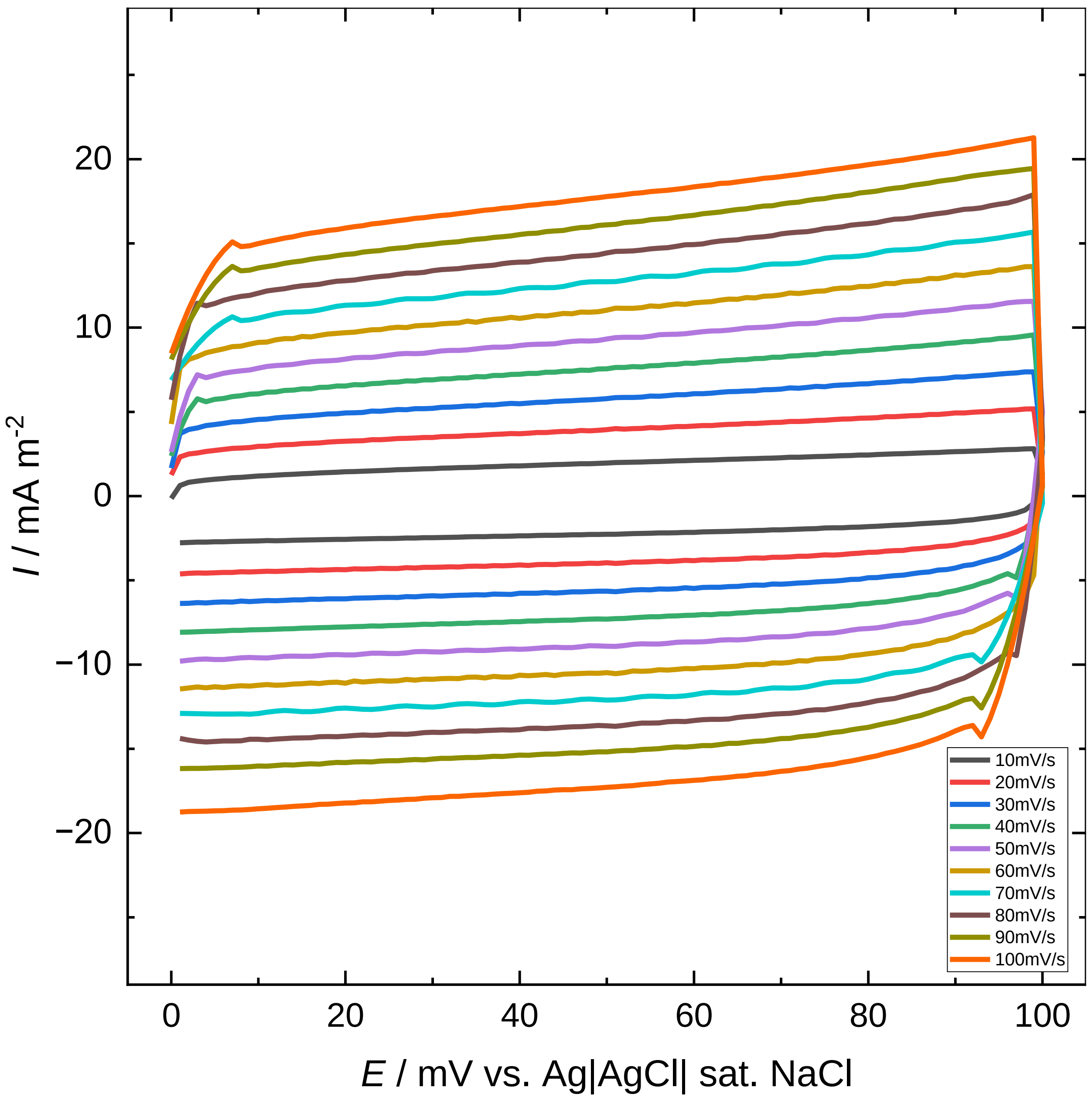

Figure S3. CV of AuEL with varying scan rate.

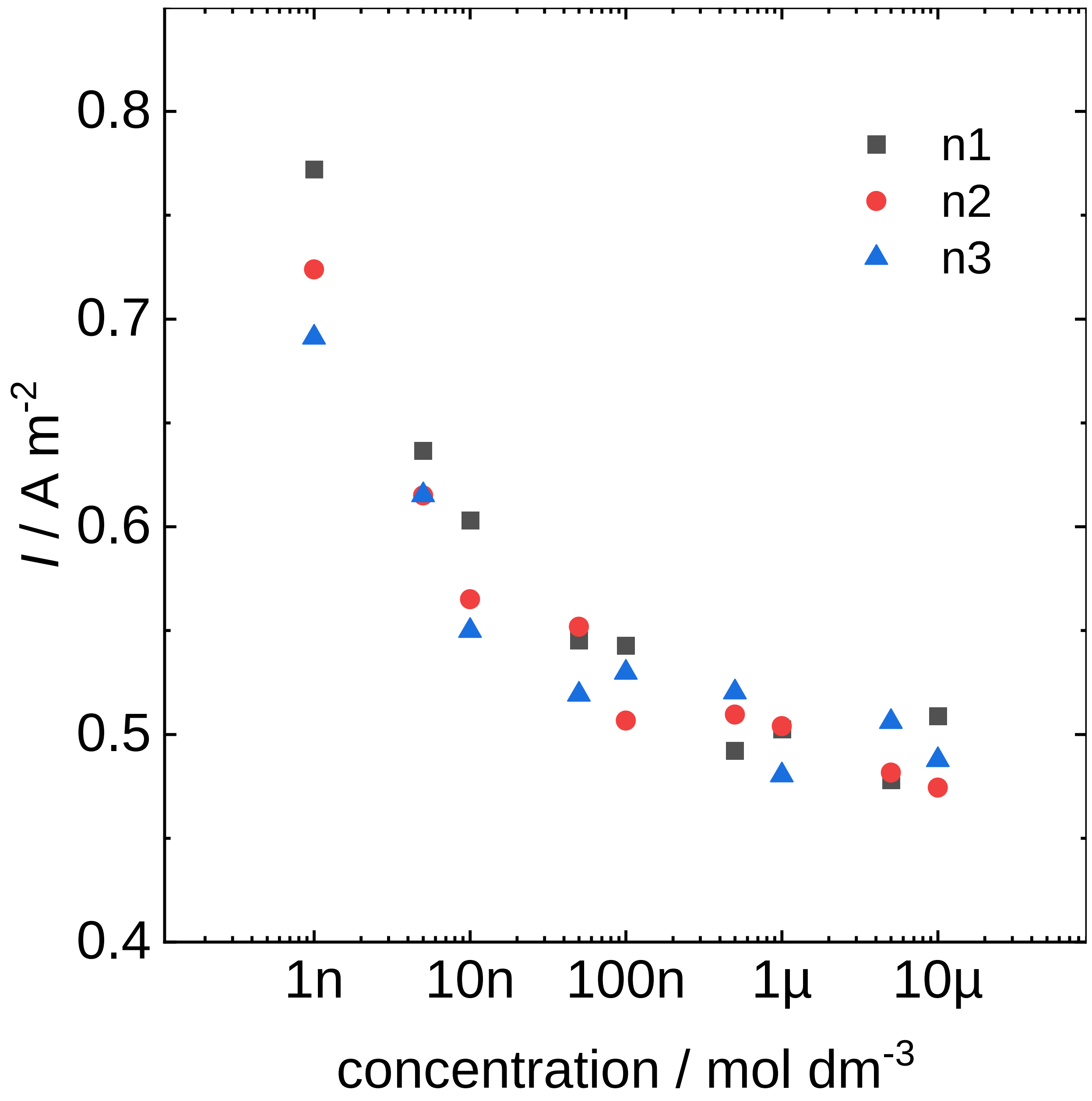

Figure S4. Each oxidation potential peak of cyclic voltammetry of 0-10 μM progesterone using P4AuEL is plotted.
